# Macrophages/Microglia Represent the Major Source of Indolamine 2,3-Dioxygenase Expression in Melanoma Metastases of the Brain

**DOI:** 10.3389/fimmu.2020.00120

**Published:** 2020-02-05

**Authors:** Dayana Herrera-Rios, Sadaf S. Mughal, Sarah Teuber-Hanselmann, Daniela Pierscianek, Antje Sucker, Philipp Jansen, Tobias Schimming, Joachim Klode, Julia Reifenberger, Jörg Felsberg, Kathy Keyvani, Benedikt Brors, Ulrich Sure, Guido Reifenberger, Dirk Schadendorf, Iris Helfrich

**Affiliations:** ^1^Skin Cancer Unit of the Dermatology Department, Medical Faculty, West German Cancer Center, University Duisburg-Essen, Essen, Germany; ^2^German Cancer Consortium (DKTK), Partner Site Essen/Düsseldorf, Essen, Germany; ^3^Division of Applied Bioinfomatics, German Cancer Research Center (DKFZ), Heidelberg, Germany; ^4^Medical Faculty, West German Cancer Center, Institute of Neuropathology, University Duisburg-Essen, Essen, Germany; ^5^Department of Neurosurgery, Medical Faculty, West German Cancer Center, University Duisburg-Essen, Essen, Germany; ^6^Department of Dermatology, Medical Faculty, Heinrich Heine University, Düsseldorf, Germany; ^7^Medical Faculty, Institute of Neuropathology, Heinrich Heine University, Düsseldorf, Germany

**Keywords:** melanoma, brain metastases, IDO, immune checkpoint molecules, tumor-associated macrophages, immunogenic microenvironment

## Abstract

The manifestation of brain metastases in patients with advanced melanoma is a common event that limits patient's survival and quality of life. The immunosuppressive properties of the brain parenchyma are very different compared to the rest of the body, making it plausible that the current success of cancer immunotherapies is specifically limited here. In melanoma brain metastases, the reciprocal interplay between immunosuppressive mediators such as indoleamine 2, 3-dioxygenase (IDO) or programmed cell death-ligand 1 (PD-L1) in the context of neoplastic transformation are far from being understood. Therefore, we analyzed the immunoreactive infiltrate (CD45, CD3, CD8, Forkhead box P3 [FoxP3], CD11c, CD23, CD123, CD68, Allograft Inflammatory factor 1[AIF-1]) and PD-L1 with respect to IDO expression and localization in melanoma brain metastases but also in matched metastases at extracranial sites to correlate intra- and interpatient data with therapy response and survival. Comparative tissue analysis identified macrophages/microglia as the major source of IDO expression in melanoma brain metastases. In contrast to the tumor infiltrating lymphocytes, melanoma cells *per se* exhibited low IDO expression levels paralleled by cell surface presentation of PD-L1 in intracranial metastases. Absolute numbers and pattern of IDO-expressing cells in metastases of the brain correlated with recruitment and localization of CD8^+^ T cells, implicating dynamic impact on the regulation of T cell function in the brain parenchyma. However, paired analysis of matched intra- and extracranial metastases identified significantly lower fractions of cytotoxic CD8^+^ T cells in intracranial metastases while all other immune cell populations remain unchanged. In line with the already established clinical benefit for PD-L1 expression in extracranial melanoma metastases, Kaplan-Meier analyses correlated PD-L1 expression in brain metastases with favorable outcome in advanced melanoma patients undergoing immune checkpoint therapy. In summary, our data provide new insights into the landscape of immunosuppressive factors in melanoma brain metastases that may be useful in the implication of novel therapeutic strategies for patients undergoing cancer immunotherapy.

## Introduction

The recent clinical success of cancer immunotherapies in patients suffering from malignant melanoma and other cancer types has revolutionized the therapeutic landscape of metastatic cancer. A major breakthrough has been achieved by the release of T cells from a suppressive “immune checkpoint,” thereby allowing effective anti-tumor responses (1–3). Numerous clinical studies in metastatic cancer, including malignant melanoma, demonstrate high efficacy and manageable toxicity by using FDA-approved immune checkpoint inhibitors against the cytotoxic T lymphocyte antigen-4 (CTLA-4) and/or programmed cell death 1 (PD-1)/PD-1 ligand (PD-L1) axis; thus, immunotherapy has rapidly become a standard treatment modality in oncology. Recent data correlated clinical benefit of PD1/PD-L1 immune checkpoint inhibition with the expression level of membrane-associated PD-L1 on tumor cells, commonly induced by Interferon-γ (IFN-γ)-mediated signaling (3). Interestingly, lymphocytes of the tumor microenvironment (TME) represent the major source of IFN-γ secretion (4–6). IFN-γ is also a strong inducer of indoleamine 2, 3-dioxygenase (IDO), an enzyme initiating the first and rate-limiting step of tryptophan degradation along the kynurenine pathway (7–9). In 2014, preclinical data identified IDO for its mechanistic synergy with immune checkpoint inhibitors (10). IDO was shown to be a facilitator of cancer development by its role to exert a strong immuno-suppressive effect through local inhibition of T lymphocytes or other immune cells, consequently contributing to tumor-protective immune suppression (11). It directs survival of CD4-positive T-helper cells and promotes regulatory T-cell differentiation (12). IDO is expressed in certain types of immune cells as well as in cancer cells, contributing substantially to immune evasion in the tumor microenvironment. However, it's “mode-of-action” is best characterized and understood in dentritic cells (13). Expression of IDO in primary melanomas and sentinal lymph nodes was identified as an independent negative prognostic factor for overall and relapse-free survival in melanoma patients (14–16). Interestingly, early clinical trials using the IDO inhibitor epacadostat in combination with immune checkpoint inhibitors targeting CTLA-4 (nivolumab) or PD-1 (ipilimumab or pembrolizumab) have reported higher response rates and longer progression free survival (PFS) when compared with checkpoint inhibitors alone (17, 18). However, recent data from a first phase III trial in patients with unresectable stage III or IV melanoma receiving epacadostat plus pembrolizumab or placebo plus pembrolizumab showed no clinical improvement for the addition of the IDO inhibitor to pembrolizumab (19). Nevertheless, efficient analyses of IDO downstream targets are lacking, as well as detailed validation trials addressing drug dosing, and therefore the usefulness of IDO inhibitors to enhance the efficacy of anti-PD-1 therapy remains unclear.

Since checkpoint inhibitors do not have to cross the blood-brain barrier (BBB) to execute activity and their effects extend over prolonged periods, potential clinical efficacy in the central nervous system (CNS) has been discussed. Metastasis to the brain is still a clinically challenging issue that may develop in up to 40% of patients with advanced disease (20) and metastatic spread is responsible for about 90% of cancer-related deaths across all entities (21). The incidence of brain metastases (BM) is rising partly due to improved visualization and diagnosis techniques but also caused by further development in systemic treatment approaches directing prolonged survival of cancer patients (22). Treatment options targeting established metastases in the CNS are rather limited, mainly caused by inefficient drug penetration across the BBB. Moreover, patients with BM are commonly excluded from clinical trials, including those investigating novel targeted therapies, as the limited survival associated with BM prevents reaching study endpoints. A multitude of cohort studies identified cutaneous melanoma as the third most common cause of BM development (23). BMs in malignant melanoma patients is frequent during disease progression, dominating prognosis and quality of life of affected patients (24–26). The incidence of overt BM at first presentation is about 20%, in advanced melanoma patients around 50% and even higher as autopsy studies reported frequencies of 55 up to 75% (27). Patients with BM from melanoma have a poor prognosis, resulting in median overall survival of 17–22 weeks (28, 29). In consequence, in 2017 the significance of BM presence was incorporated into the American Joint Committee on Cancer (AJCC) staging system as an independent prognostic factor in patients with malignant melanoma (30).

The understanding of the brain as an “immune-privileged” organ has recently changed due to detailed characterization of border-associated structures connecting the CNS with the periphery. Thus, in 2015 a functional draining lymphatic vascular system of the CNS has been described for the first time by different groups implicating the transport of brain-specific antigens into cervical lymph nodes (31, 32). Nevertheless, the entry of the CNS is strictly controlled by the BBB to protect the brain from neurotoxic mediators, but patrolling leukocytes such as CD4^+^ and CD8^+^ T cells and bone marrow-derived antigen-presenting DC have already been identified in the meninges and choroid plexus in pre-clinical models and men (33, 34). Thus, some BM resected under ipilimumab therapy showed dense infiltration of CD8^+^ cytotoxic tumor infiltrating lymphocytes (TILs) and FoxP3^+^ regulatory T cells, indicating a triggered immune response under therapy (35). The immunosuppressive properties of the brain parenchyma, which is highly divergent compared to the rest of the body (36–38) could therefore strongly impact any local anti-tumor response. As such, the reciprocal interaction between tumor and immune cells as well as the association between the density and localization of lymphocytic infiltrates in melanoma BM is currently under investigation. Early results from ongoing trials indicate promising activity of immune checkpoint inhibitors by using anti-CTLA-4 (39), anti-PD-1 (40, 41) or a combination of both therapies (42) also in the CNS. Although intracranial response rates up to 47 % were achieved, this response was not translated into improved patients survival (42). As such, it has become clear, that neoplastic processes in the brain may induce prominent anti-tumor immune response. In consequence, IDO could function as a suitable target to enhance the efficacy of checkpoint therapy in the brain. However, the immunosuppressive mechanisms in BM are far away from been understood. Therefore, a deeper understanding of the cellular composition of the BM-associated TILs and its impact on immunosuppressive factors is necessary for developing novel therapeutic combination strategies against BM establishment and outgrowth. Nevertheless, the impact of IDO expression in the presence of tumor-infiltrating lymphocytes (TILs) and other immunoreactive inflammatory cells as macrophages/microglia or dendritic cells for the responsiveness to cancer immunotherapy is still elusive.

Thus, here we provide the landscape of IDO expression in coevolution with the immunogenic microenvironment in a large cohort of melanoma patients with BM, including patients with matched pairs of BM and extracranial melanoma metastases to correlate intra- and interpatient data with therapy response and survival.

## Materials and Methods

### Patients and Patient-Derived Tissue Samples

We analyzed formalin-fixed and paraffin-embedded (FFPE) tissue samples from metastases of 72 patients with BM from malignant melanoma. For 19 patients, matched pairs of BM and metastases at extracranial sites were available that allowed for intra-individual comparative analyzes. In total, we included 74 intracranial and a set of 22 matched extracranial melanoma metastases in our study. Relevant clinical data of these patients are listed in Table 1. The cohort was collected as part of the “*Brain_Prevent*”consortium in Germany, including following sites: Department of Dermatology, Institute of Neuropathology, Department of Neurosurgery, all Essen and the Institute of Neuropathology and Department of Dermatology at the Heinrich-Heine University Düsseldorf, all Germany. In detail, tissue samples from intracranial melanoma metastases were retrieved from the tissue banks at the Institute of Neuropathology, University Hospital Essen, and the Institute of Neuropathology, Heinrich Heine University Düsseldorf, Germany. Extracranial metastases of corresponding patients (“matched-pair” samples) were provided by the Skin Cancer Biobank (SCABIO) of the Department of Dermatology, University Hospital Essen, or the Department of Dermatology, Heinrich Heine University Düsseldorf, Germany. All intracranial and extracranial melanoma metastases were histopathologically diagnosed (ST-H, TS, JR, KK, GR). Clinical data and follow-up information were obtained from the SCABIO or the West German Biobank (WBE) of the University Hospital Essen. Informed patient consent was obtained from all patients. The study was performed with approval by the ethics committee of the Medical Faculty, University Duisburg-Essen (ethics approvals no. 11-4715 and no. 15-6723-BO), and the ethics committee of the Medical Faculty, Heinrich Heine University Düsseldorf (ethics approval no. 5246).

**Table 1:** Patients characteristics and clinical data.

**Characteristics**		
**Patients**, ***n***		72
**Matched-pair**, ***n***		19
**Metastasis**, ***n***		
Intracranial		74
Extracranial		
Skin		19
Adrenal gland		2
Lymph node		1
**Gender**, ***n***	**Age at first BM diagnosis (years** **±** ***SD*****)**	**Age at BM surgery (years** **±** ***SD*****)**
Female, 34	58 ± 14	58 ± 13
Male, 38	59 ± 15	59 ± 14
**Therapy**, ***n*** **patients (%)**		
Mono-CT		5 (6.9)
Mono-RT		7 (9.7)
Mono-IMT		2 (2.8)
CT+RT		12 (16.7)
CT+IMT		6 (8.3)
RT+IMT		6 (8.3)
CT+RT+IMT		13 (18.1)
Unknown		21 (29.2)
**Number of brain metastasis, patients (%)**		
1		53 (73.6)
2		9 (12.5)
3		6 (8.3)
4		2 (2.8)
5		1 (1.4)
6		1 (1.4)
**Location of intracranial melanoma metastases**, ***n*** **=** **patients (%)**		
Cerebrum		48 (16)
Cerebellum		6 (8)
Unknown		18 (25)
**Clinical outcome**, ***n*** **(%)**		
Alive		16 (22.2)
Dead		37 (51.4)
Unknown		19 (26.4)

*CT, chemotherapy; RT, radiotherapy; IMT, immunotherapy*.

### Immunohistochemistry

Serial sections were prepared from formalin-fixed, paraffin-embedded tumor biopsy samples. Standard hematoxylin and eosin (H&E) staining was performed for visualization of the tissue morphology. For each biopsy the tumor area was marked as “Region Of Interest (ROI)” by the neuropathologist or the dermatopathologist. Immunohistochemistry was performed using primary antibodies against the following proteins: IDO (clone D5J4E, Cell Signaling Technology, Frankfurt am Main, Germany), CD45RO (clones 2B11 + PD7/26, Dako, Denmark), CD3 (clon SP7, DCS Innovative Diagnostik Systems, Hamburg, Germany), CD8 (clone C8/144B; Dako, Denmark), Foxp3 (clon 206D, BioLegend, Koblenz, Germany), PD-L1 (clone E1L3N, Cell Signaling Technology, Frankfurt am Main, Germany), AIF1 (Acris, Hamburg, Germany), and CD11c (clon 5D11, DCS Innovative Diagnostik Systems, Hamburg, Germany), CD68 (clone PG-M1, Dako, Denmark), CD23 (clone 1B12, Novocastra, Wetzlar, Germany), CD123 (clone 6H6, Abcam, Newcastle, UK). Staining was performed by using the Dako REAL detection system and the goat-on-rodent AP-polymer Kit (GAP514H, Biocare medical, Zytomed) on the Dako Autostainer 46 System followed by hematoxylin counterstaining (Dako, Denmark). To avoid staining specific variations, all sections per individual marker were stained in the same run on the autostainer. Slides were digitalized using Amperio AT2 (Leica Biosystems Imaging INC) at the WBE.

### Quantitative Digital Pathology/Tissue Image Analysis

Protein expression analyses on a cell-to-cell basis was performed by using the Definiens Tissue Studio Software® (Definiens AG, München, Germany). Intratumoral analyses of each sample were made by using the marked ROI (tumor area) and this ROI was transferred to each individual staining per tissue sample for further histopathology-based analyses. Peritumoral analyses were made by analyzing the individual markers at the tumor margin of the stroma as already described (43). For each protein, individual parameters were established by using the corresponding IgG control for each primary antibody. We generated two tissue sections on each slide which have been used to stain in parallel the IgG control and the primary antibody on the same section and in the same run of the Dako Autostainer. The “background” intensity given by the IgG control was used as threshold for each individual maker. For IDO expression level analysis we calculated the thresholds for following individual categories on the basis of the calculated mean: low (0.05), moderate (0.09) and high (0.3). The threshold for CD45 (0.03), CD3 (0.03), CD8 (0.1), Foxp3 (0.1), AIF (0.07), and CD11c (0.07) was calculated by discriminating false positive detection given by melanophages which we excluded by using the corresponding H&E sections. Areas without nuclei in between the tumor area (wholes, cuts, punch biopsies) were excluded in order to calculate the individual number of positive cells per total number of tumor cells.

### Statistical Analysis

All statistical analyses were performed in R version 3.2.3. Survival analysis was calculated using the R packages survival (2.41–3) and survminer (0.4.3). The end of follow-up period of the study was December 2017. Two clinical survival outcome endpoints were chosen for the endpoints analysis: Overall Survival (OS) and Progression-Free Survival (PFS). The OS period was calculated from the date of initial diagnosis until the date of death from any cause. PFS was identified by using the period of time after date of initial melanoma diagnosis until the development of a brain metastasis. For univariate analysis, long-rank *p*-values were calculated. For multivariate analysis, Cox's proportional hazards models were used. Plots were generated using the ggplot2 (2.2.1). Multivariate Cox proportional hazards regression models were fit using function *coxph* and the forest plots were generated using the *ggforest* command. The Wilcoxon paired test was used to calculate the correlation of the infiltrates of immune cells in patient-matched brain and skin biopsies. A *p*-value correction was applied using the “holm” method. An adjusted *p*-value of 0.1 was considered significant. Spearman correlation was performed to check the relationship of total IDO expressing cells in ICM and ECM to the PD-L1 expression (intensity) status. Plots were drawn using ggplot2 package in R. The curve was smoothened using a linear regression (*lm*). A *post-hoc* Tukey HSD (Hosnest Significant Difference) followed by Anova was performed to test the pairwise correlation among the PD-L1 expression values and IDO states (total IDO expressing cells; high, medium and low intensity of IDO-positive cells).

## Results

### Patient Cohort

In total, our study included 72 patients, 34 women, and 38 men, with an age of 58 ± 13 and 59 ± 15 years (mean ± SD), suffering from malignant melanoma and diagnosed for the development of brain metastases (for detailed description of the patient characteristics see Table 1). From 19 of these 72 patients “matched” biopsies were available from extracranial sides, thus allowing for intrapatient analyses. Out of 74 intracranial melanoma metastases from the 72 patients, 48 metastases were located in the cerebrum and six tumors were resected from the cerebellum, while information on supra- vs. infratentorial location was missing for 18 BM. The set of 22 “patient-matched” extracranial metastases from 19 patients included 19 cutaneous, two lymph node and one adrenal gland melanoma metastases (Table 1).

### Distinct IDO Expression Patterns in Metastases of Malignant Melanoma

**Figure 1:**
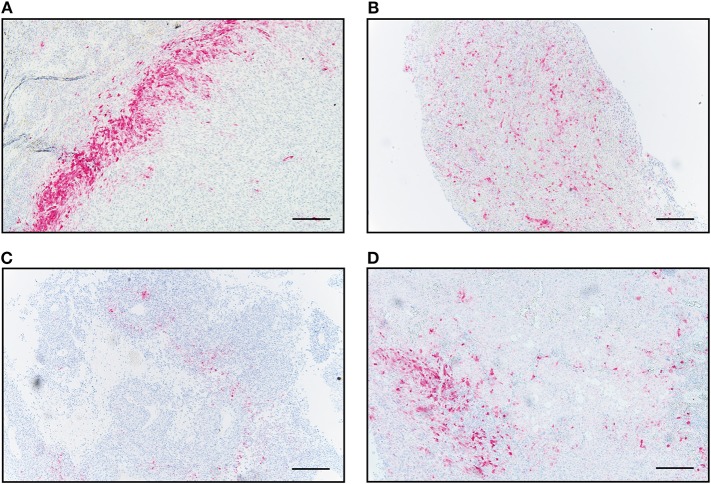
Immunohistochemical and pathological analyses of IDO distribution in human melanoma metastases. Four distinct infiltration patterns of IDO-positive cells were predominantly detected independent of intracranial or extracranial origin. Representative images for the individual distribution patterns are presented in intracranial metastases. IDO-positive cells in a **(A)** “border-like,” **(B)** “diffuse,” **(C)** “partial rim” and **(D)** combined “partial rim plus diffuse” localization. Scale bar, 200 μm.

First, we detected cytoplasmic IDO expression in all 74 intracranial and 22 extracranial metastases of advanced melanoma patients (Figure 1). Interestingly, we observed distinct patterns of IDO tissue distribution. One expression pattern we defined as “border-like” due to the exclusive location of IDO-positive cells at the invasive tumor-stroma interface, surrounding the tumor like a wall (Figure 1A). This pattern was detected in 3/74 (4%) intracranial and 4/22 (18.1%) extracranial metastases. The second expression pattern which we named “diffuse” was frequently seen in both metastatic tissue sites, i.e., was present in 59/74 (80%) intracranial and 8/22 (36.3%) extracranial metastases. This pattern corresponded to a widespread diffuse occurence of IDO^+^ cells in the tumor mass (Figure 1B). The third pattern, which we described as “partial rim,” corresponded to an interrupted border-like expression (Figure 1C). This pattern was found in 5/74 (7%) intracranial and 6/22 (27.3%) extracranial metastases. A fourth pattern combined the “partial rim” and the “diffuse” pattern and was detected in seven metastases of the CNS (9%) and 4 cases of extracranial sites (18.1%, Figure 1D).

### Intratumoral Variability of IDO Expression Level Mediate PD-L1 Surface Expression

**Figure 2:**
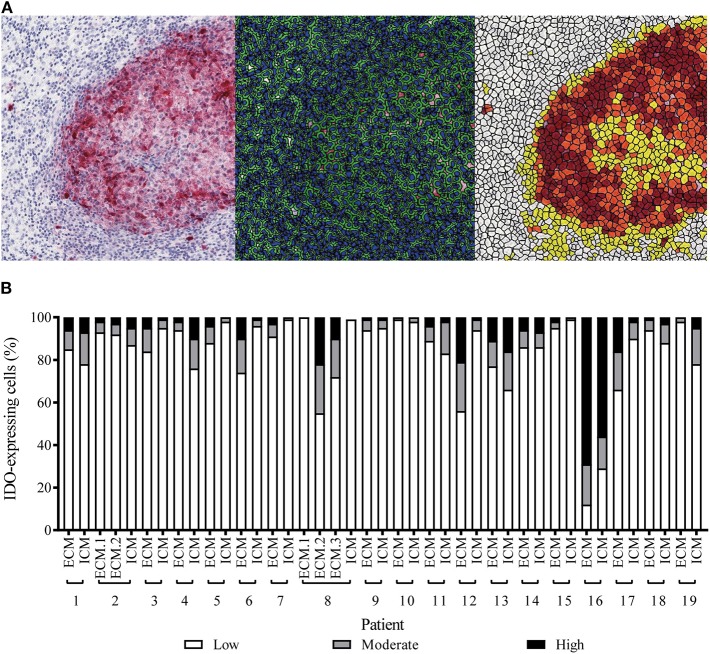
Quantitative assessment of IDO-expression intensity in patient-matched melanoma metastases of intracranial and corresponding extracranial origin. **(A)** Representative images for immunohistochemical-based IDO-expression (left), parameter-based separation of cell-cell borders (middle), classification of high (brown), moderate (orange) and low (yellow) expression intensities or IDO-negative areas (white, right). **(B)** Statistically-based calculation for the intratumoral percentage of high (black), moderate (gray), low (white) IDO-expressing cells in intracranial (ICM) or extracranial (ECM) metastases of individual melanoma patients (*n* = 19).

In addition to the distinct patterns of IDO immunopositivity in malignant melanoma metastases, we detected also an intratumoral heterogeneity for the IDO expression intensity, independent of the tissue origin (Supplementary Figure 1). By using quantitative digital pathology tissue diagnostics, we generated an individual cell-by-cell threshold for the immunohistochemistry-based IDO intensity level (Figure 2A). By using the “patient-matched” cohort of 19 patients, we detected—with exception of patient no. 16—that more than 50% of the IDO^+^ tumor area was represented by melanoma cells expressing low levels of IDO and that only 10–20% of IDO^+^ tumor area was represented by immune cells, which showed moderate or high expression intensity (Figure 2B). However, Kaplan-Meier analysis revealed that neither the IDO expression level nor the total number of IDO-positive cells in the distinct metastases impacted disease progression or survival of advanced melanoma patients (data not shown).

We next asked the question whether the immunosuppressive factor IDO directs the expression of other immunosuppressive molecules with regard to the PD-1/PD-L1 axis. Tukey HSD test was performed to test for significance. We found that only the tumor cell-associated IDO, represented by low IDO intensity, strongly correlates with PD-L1 surface expression (*p* = 0.0006) and, in consequence, that the number of IDO^+^ tumor cells directs the intratumoral expression level of the immunosuppressive molecule PD-L1 (*p* = 0.00015, Table 2).

**Table 2:** Correlation of PD-L1 and IDO.

**Number of cells**	**Adjusted *p*-value**
Total IDO / PD-L1	0.00015
Low IDO / PD-L1	0.00066
Moderate IDO / PD-L1	0.11716
High IDO / PD-L1	0.06842

*A post-hoc-Tuckey HSD (Honest Significant Difference) followed by anova was performed to test the pairwise comparisons among the PD-L1 expression values and IDO (Total number of IDO expressing cells, high, medium, and low IDO expressing cells). The 95% confidence for lower and upper intervals is mentioned together with the adjusted p-values*.

### IDO Favors an Immunosuppressive Signature in Melanoma Brain Metastases Directing Efficacy of Cancer Immunotherapy

**Figure 3:**
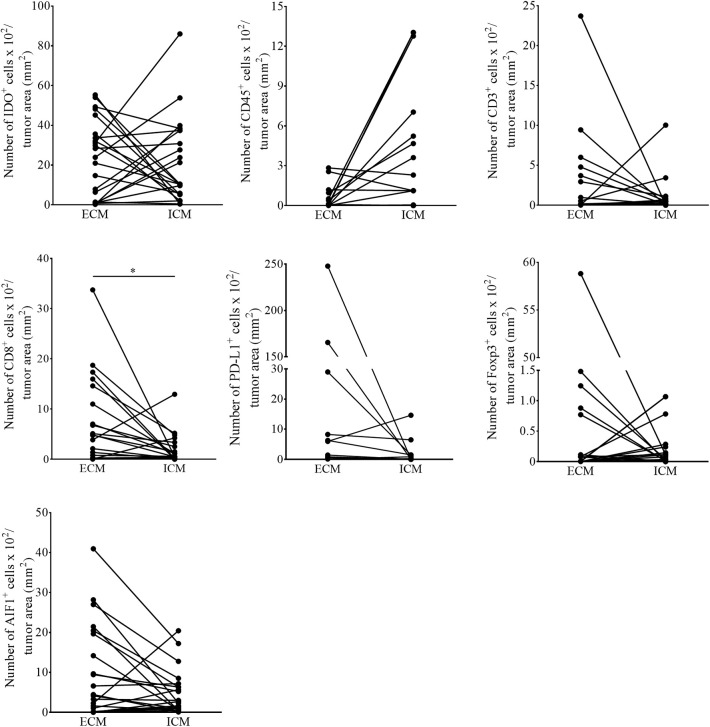
Quantitative assessment for the number of immunoreactive cells in patient-matched melanoma metastases. The number of IDO, CD45, CD3, CD8, FoxP3, PD-L1, and AIF1 positive cells in intra- (ICM) and corresponding extracranial (ECM) metastases of individual melanoma patients (*n* = 19 patients, *n* ECM = 22, *n* ICM =19; *p < 0.05).

**Figure 4:**
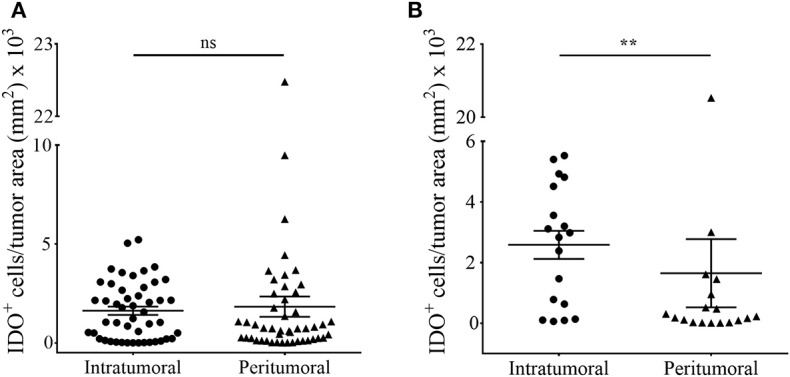
Comparative analyses of intratumoral and peritumoral IDO-expression in human melanoma metastases of intracranial and extracranial origin. The total number of IDO-positive cells localized in the tumor (intratumoral) or around (peritumoral) in **(A)** ICM and **(B)** ECM was quantified in accordance to histopathological labeling of the tumor area by using the quantitative digital pathology tissue analysis system Definiens Tissue Studio (*n* ICM = 48/47 patients, *n* ECM = 18/16 patient, *n* matched-pairs = 16; **p < 0.05). To ensure statistical balance between both parameters we excluded all patient samples from the analyses in the case of missing stroma in the individual tissue specimen.

The current knowledge and understanding of cancer immunotherapy has changed dramatically during the last decades. Multiple clinical data let assume that the amount and also the localization of the lymphocytic infiltrate in different cancer entities directs the response to cancer immunotherapy. In 2014, Tumeh and colleagues could show that pre-existing CD8^+^ T cells distinctly located at the invasive tumor front correlate with the expression of the immunosuppressive checkpoint molecules PD-1/PD-L1, predicting response to immunotherapy in patients suffering from malignant melanoma (3). Because the detailed localization of IDO^+^ cells in melanoma metastases of the brain and its impact on the recruitment of TILs is still elusive, we addressed this issue in our cohort of melanoma BM and matched extracranial melanoma metastases. We first called intra- and interpatient analyses by using our patient-matched cohort for the number of cells expressing IDO and markers of the lymphocytic infiltrate (CD45, CD3, FoxP3, CD8), PD-L1 and the Allograft inflammatory factor 1 (AIF-1), mainly expressed by macrophages/microglia. We detected a significant higher number of CD8^+^ T cells in metastases of extracranial sites when compared to metastases of the CNS (*p* = 0.016), whereas all other markers remained unchanged represented (Figure 3). Interestingly, we found that the localization of IDO-positive cells is strongly paralleled with the localization of the lymphocytic infiltrate, with exception of FoxP3-positive regulatory T cells, which were also recruited into the tumor mass, but not localized in areas of high IDO-expression as exemplarily presented in Supplementary Figure 2 in cutaneous melanoma metastases. Moreover, whereas we detected a balanced expression of IDO^+^ cells in metastases of the brain (*p* = 0.351, Figure 4A) we found significantly higher fractions of IDO-expressing cells with intratumoral localization in extracranial metastases when compared to the peritumoral microenvironment (*p* = 0.005, Figure 4B).

The development of brain metastases is a significant cause of morbidity or mortality for patients with metastatic cancer, including melanoma. However, for still unclear clinical reasons some patients show a better outcome as others. By using a total of 38 cases, 13 patients who received standard care therapy and 17 cases which received immune checkpoint inhibitors alone or in combination with other therapies, showed a median survival of 228 vs. 336 days using the time of first BM observation and date of death. Therefore, we asked whether the expression of IDO itself, independent of the cellular source, is associated with the recruitment of tumor infiltrating lymphocyte subsets and whether this immunoreactive infiltrate influences the clinical outcome of melanoma patients in our cohort.

**Figure 5:**
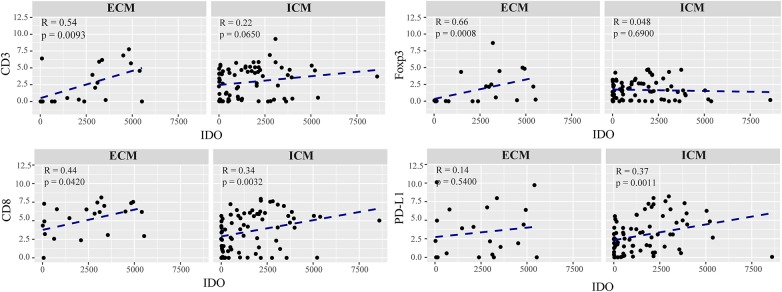
Correlation of the immunoreactive infiltrate and IDO expression in extracranial and intracranial melanoma metastases. Each dot in the scatter plot represents an individual patient. The x-axis represents the total number of IDO expressing cells and the y-axis shows the expression of CD3, FoxP3, CD8, and PD-L1 represented in a logarithmic scale. ECM and ICM denotes the exracranial and intracranial melanoma metastases. Spearman correlations were performed and regression was calculted using (*lm*) function.

**Figure 6:**
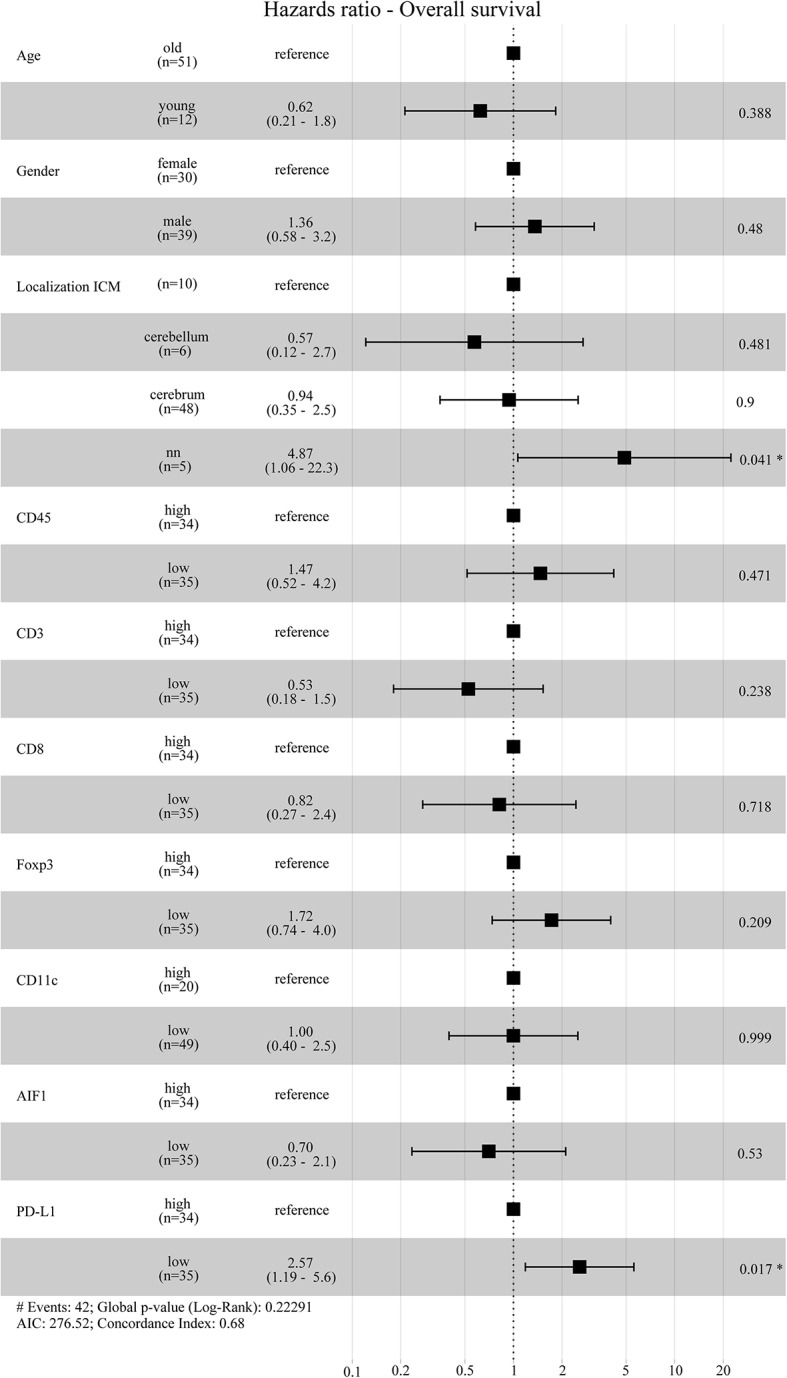
Hazard ratio Overall survival. Forest plot for the cox proportional hazards model was calculated by using age, gender, localization of the ICM and immunoreactive infiltrates. The patients below 40 years of age at death were grouped in the “young” group and vice versa. For the immune cell infiltrates the patients were grouped into a “high” or “low-group” based on the median expression values. According to the multivariate model, low PD-L1 expressing patients have a significantly higher hazards ratio and thus poor overall survival compared to patients with high PD-L1 expression.

We detected a strong correlation between IDO positivity and infiltration of CD8^+^ cytotoxic T cells in intra- (*R* = 0.34, *p* = 0.0032) and extracranial (*R* = 0.44, *p* = 0.0420) metastases, whereas expression of IDO paralleled by the recruitment of regulatory T cells, as evidenced by CD3/FoxP3 immunostaining, was exclusively seen in metastases at extracranial sites (*R* = 0.66, *p* = 0.0007, Figure 5). However, preforming a multivariate Cox proportional hazards regression model we did not observe a significant association for disease progression with regard to the individual lymphocyte subtypes in metastases of the brain (Supplementary Figure 3). Interestingly, statistical analyses identified a significant positive correlation of IDO with PD-L1 expression which was solely detectable in metastases of intracranial sites (*R* = 0.37, *p* = 0.0011) predicting worse prognosis in these patients in the multivariate analyses (*p* = 0.017, Figures 5, 6).

**Figure 7:**
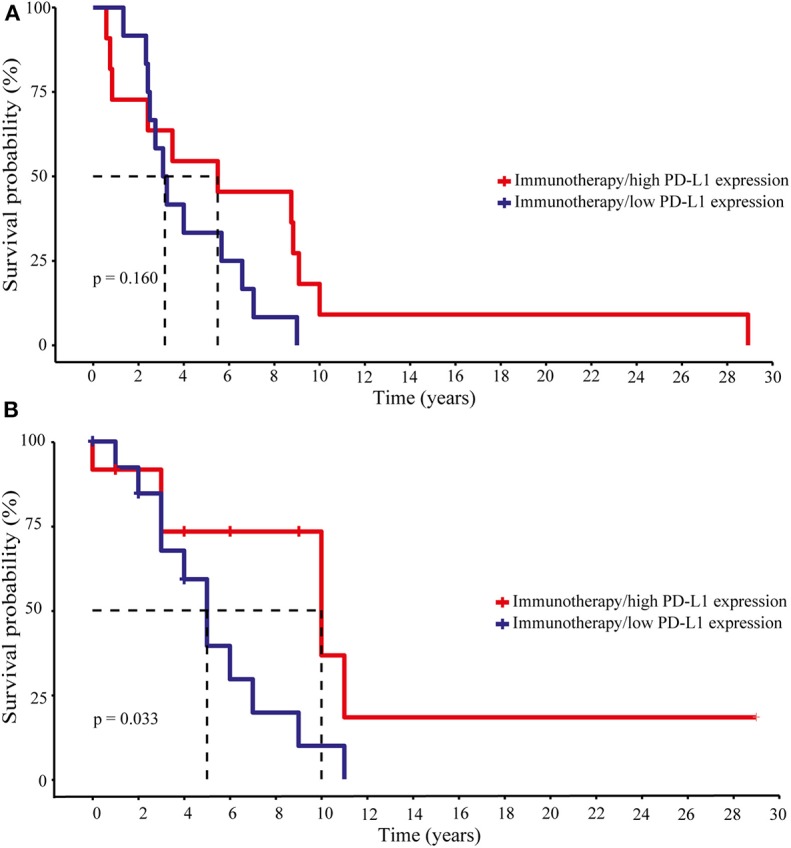
Disease progression and survival analyses of melanoma patients under immunotherapy with respect to intracranial PD-L1 expression. Patients were divided into two groups, “high” and “low” PD-L1 expression due to the median PD-L1 expression level. **(A)** Progression-free survival. Long-rank test statistics show no differences in the progression-free survival for patients with high and low PD-L1 expression (Log-rank *p*-value 0.160). **(B)** Overall survival. According to the Kaplan–Meier curve patients with high PD-L1 had a greater benefit from immunotherapy and showed a better overall survival (Log-rank *p*-value 0.033). Dotted lines indicate the 50% survival probabilities for both groups.

The recent success of cancer immunotherapy in different cancer entities by using the so called “checkpoint inhibitors” paralleled the expression of intratumoral PD-L1 with clinical response (3, 44). Thus, we analyzed whether patients with melanoma BM receiving checkpoint inhibitors as a monotherapy (*n* = 2) or in combination with chemotherapy (*n* = 6), radiotherapy (*n* = 6) or both (*n* = 13) at any time of disease might gain a clinical benefit from high PD-L1 expression in their brain metastases. A Kaplan-Meier survival analysis was performed and the patients were divided in two groups based on median PD-L1 expression. Whereas, the expression of the immune checkpoint molecule PD-L1 did not appear to have an impact on disease progression (log-rank *p* = 0.16, Figure 7A) it significantly affected patients survival (log-rank *p* = 0.033, Figure 7B). The 50% survival probabilities for the patients with low PD-L1 expression is 5 years whereas, patients with a high PD-L1 expression showed a 50% survival probability of 10 years. However, due to the limited number of patients, these results must be validated in a larger cohort of advanced melanoma patients with brain metastases undergoing checkpoint therapy.

### High IDO Expression Level Are Primarily Represented by Macrophages/Microglia

**Figure 8:**
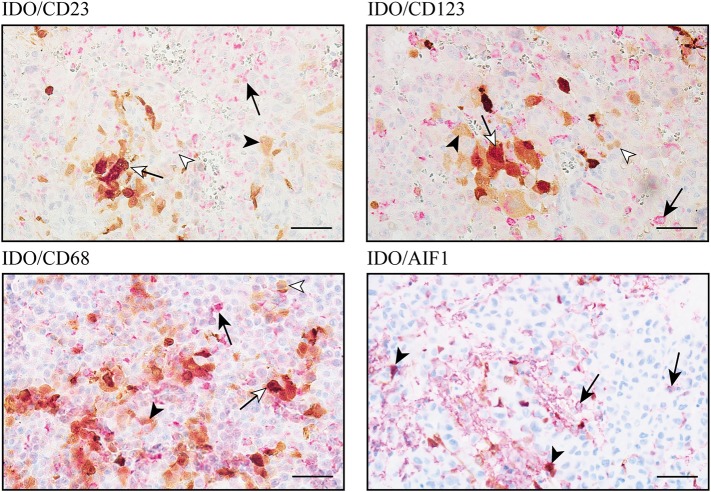
IDO-expression on cellular components of the immunoreactive tumor infiltrate in melanoma metastases of the central nervous system. Immunohistochemical-based co-immunostaining for IDO (brown) and indicated makers for subpopulations of DC and macrophages/microglia (all in red) exemplarily shown in intracranial (right) metastases. Black arrowhead: IDO^+^ macrophage; black arrow: single expression of the indicated markers (CD23, CD123, CD68, or AIF1); white arrow: co-expression of IDO plus indicated maker in macrophages, white arrowhead: single marker detection. Representative images were presented. Scale bar = 50 μm.

In addition to tumor cells, expression of the immunomodulatory protein IDO by subpopulations of tumor-associated immune cells, e.g., dendritic cells, macrophages and B-lymphocytes, has been reported in different types of cancer (45–47). However, the major cellular source of IDO expression in intracranial melanoma metastases is still unknown. Since we found low IDO expression levels in melanoma cells of BM, we went further into the analysis of distinct subsets of monocytes by co-staining experiments including 10 selected cases each from our “matched-pair” cohort of patients with tissue from intracranial and extracranial melanoma metastases. To avoid false positive detection mediated by brownish melanophages or melanocytic tumor cells, we exclusively selected amelanotic tissue samples for these analyses. First, we addressed the expression of IDO in different subtypes of DCs by using specific antibodies against the integrin a-x (CD11c), the Fcε-Rezeptor II (CD23) expressed on follicular DC and the interleucin 3 receptor (CD123), represented in conventional DCs (cDCs) and plasmacytoid dendritic cells (pDCs) as presented in Figure 8. Nevertheless, it is important to note that all of these markers are also expressed by different subpopulations of monocytes and granulocytes, dependent on their level of maturation and activation. Intratumoral CD11c expression was limited to brain metastases whereas only two cases showed co-expression of CD11c with IDO. We detected IDO^+^/CD23^+^ co-expression in 1/10 intra- and 2/10 extracranial metastases. Finally, IDO^+^/CD123^+^ double-positive cells could be detected in 9/10 brain metastases but only in 3/10 metastases at extracranial sites. Interestingly, IDO-positive CD23 and CD123 cells were histopathologically confirmed as macrophages. Double immunostaining for IDO with CD68, a protein that is highly expressed by cells of the monocyte lineage and tissue macrophages, or AIF-1, identified a strong infiltration by IDO^+^ macrophages/microglia in all analyzed metastases independent of the tissue origin. In detail, 37 ± 2% (mean ± SD) or 48 ± 11% (mean ± SD) of CD68^+^ macrophages and 17 ± 8% (mean ± SD) or 11 ± 3% (mean ± SD) of AIF1^+^ macrophages/microglia co-expressed IDO in metastases of intracranial or extracranial sites, and presented high expression level by using the individual thresholds for IDO determined by the Definiens pathology software (Figure 8). Although the expression intensity of IDO in DCs subpopulations was comparable to that in macrophages/microglia, it became clear that the macrophage/microglia population in melanoma metastases is of greater importance due to the very limited presence of DCs in the tumors of our cohort.

## Discussion

Following non-small cell lung cancer (NSCLC) and breast cancer, melanoma is the third most common origin of metastases to the brain. However, they exhibit the highest risk for cerebral tropism of all cancer entities, reflected by a 50–75% chance for development of intracranial metastases in advanced melanoma patients (27, 48, 49). Altough the local treatment approaches using whole-brain radiation therapy, stereotactic radiosurgery and/or surgical resection remain important, the use of systemic therapies has initiated a new therapeutic area in the management of melanoma brain metastases. Despite recent advances in the systemic treatment of extracranial metastases by using BRAF-targeted therapy in patients harboring BRAF^V600E^-mutant melanomas or inhibitors targeting immune checkpoint molecules, the treatment of melanoma brain metastases remains a major challenge. Multiple phase II and III studies have shown that ipilimumab and nivolumab are active in advanced melanoma and that the combination therapies involving PD-1 or CTLA-4 inhibitors presented a superior efficacy when compared to the individual monotherapies (50–53). Nevertheless, only 40–45% of melanoma patients benefit from cancer immunotherapy *per se* (51).

Although the use of immune checkpoint inhibitors targeting PD-1 and/or CTLA-4 has nowadays become an established therapy in melanoma, it is still critical to transfer our knowledge from extracranial sites to intracranial melanoma lesions with respect to the unique “immune-specialized” microenvironment of the brain (54, 55). After extravasation of tumor cells into the brain parenchyma they enter a fundamentally different tissue environment with respect to the metabolic situation, the cellular compositions, the brain-specific extracellular matrix proteins and the immunoreactive heterogenous cell population with regard to the primary site of their origin (56). This appears particularly relevant in the context of immune cell activity against extravasated single cancer cells and micrometastases when the normal brain parenchyma, including the blood-brain barrier, is still largely intact. In line with this concept, melanoma patients developed remarkable high rates of BM during Ipilimumab in one study (57), which fits to the empirical impression of many clinical experts in the field. In contrast, brain macrometastases have been found to respond well to ipilimumab and other immune checkpoint inhibitors in subsets of patients (35, 58) which supports the general concept that preventing metastatic outgrowth is very different (biologically and therapeutically) from targeting large established macrometastases. However, limited clinical data are available adressing the activation of checkpoint inhibitors in the CNS. One of the first phase II studies evaluated the activity of ipilimumab in patients with melanoma brain metastases and enrolled 72 patients in a two-arm clinical trial (39). Fifty-one patients were neurologically asymptomatic and therefore did not receive any corticosteroids at time of enrollment (arm A), whereas 21 patients showed symptomatic disease and were on a stable dose of corticosteroids (arm B). This study achieved intracranial response rates of 16 and 5% in cohort A and B, respectively, and hence confirmed the activity of anti-PD-1 therapy in the CNS, but also highlighted the importance of being off corticosteroids at the time of therapy initiation. Multiple follow-up clinical trials addressed the activity of combination therapies targeting PD-1 and CTLA-4 vs. monotherapy in advanced melanoma patients (42, 59, 60). In summary, all achieved activities at the intracranial site, albeit to limited extents. Interestingly, novel data of the multicentre open-labeled randomized phase II trial NCT02374242 suggested a higher chance of long-term durable intracranial response by using the combination of ipilimumab and nivolumab in patients with asymptomatic untreated melanoma brain metastases (42). According to the current clinical data, own unpublished data by using the primary melanoma model MT/ret, which spontaneously induces multiple cutaneous melanoma and distant organ metastases, including the CNS, show that inhibition of the PD-1/PD-L1 axis resulted in diminished intracranial tumor load but failed to suppress the establishement of micrometastases in the CNS (Helfrich, unpublished data) (61, 62). These clinical data argue for a principle ability of immune checkpoint inhibitors to reach meaningful anticancer immunity in the brain, however, the current therapies seem to fail for their suppression of metastatic seeding of the CNS by cancer cells.

IDO expression and activity has been documented in several cancer entities and has been correlated with negative prognostic factors (9). Thus, it was only a question of time until first clinical trials combined IDO inhibitors like epacadostat or navoximod with inhibitors targeting the PD-1/PD-L1 axis or CTLA-4 in differnt tumor entities, including advanced melanoma (19, 63–66). Since all of this studies demonstrated acceptable safety, good tolerability, and pharmacological activity, there was no clear evindence of patients benefit when combined to PD-1/PD-L1 inhibitors. Nevertheless, IDO data with respect to the CNS metastases are missing.

In the present study, we analyzed tissue samples of 74 intracranial metastases from 72 advanced melanoma patients and 22 matched melanoma metastases at extracranial sites from 19 of the 72 patients. We specifically adressed the expression of immunosuppressive mediators such as IDO and PD-L1 in the context of the tumor-associated immunoreactive infiltrate. We found that IDO is expressed in different patterns in melanoma brain metastases indicating IDO expression as a marker of anti-tumor immune response. First, in contrast to data described by Krähenbühl et al. (16), who analyzed different primary cutaneous melanoma types and corresponding organ metastases (with exception of CNS metastases) in 43 patients undergoing cancer immunotherapy or targeted therapy and described IDO immunoreactivity in 17/43 pretreated samples, we found that IDO expression is highly consistant indicating IDO as a marker of anti-tumor immune response. Moreover, a strong correlation of IDO expression in peritumoral sites of the primary tumors has been linked to IDO expression in the sentinel lymph node, directing the numbers of intratumoral lymphocytes as a result of immune control (14). In contrast to CNS metastases, we found higher IDO-positive cell numbers in the tumor mass when compared with peritumoral localization in melanoma metastases at extracranial sites, which would fit to an ongoing anti-tumoral immune response, since high levels of IFN-γ are secreted during this process. In addition, our investigation revealed different distribution patters of IDO-positive cells in melanoma metastases, but these were independent of the metastatic origin. Interestingly, neither the localization nor the distribution pattern of IDO had an impact on patient outcome. However, the heterogeneous expression of the immunosupressive IDO which we detected both, within and between patients, may explain the high variation in the clincal response to IDO combination treatment (19, 63, 64).

Since several studies correlated high TIL levels with favorable outcome (67–69) our data are in line with the work of Harter et al. (37). Neither disease progression nor patient survival was affected by the number of TILs in melanoma brain metastases in our patient cohort *per se*. As TILs represent also the major source for the secretion of inflammatory stimuli such as IFN-γ and TNF-α (4, 70), resulting in activation of lymphocytes and induction of PD-L1 expression, we analyzed this aspect also in our tissue specimens. Interestingly, with regard to the expression of the immunosupressive molecule IDO, we found that IDO-positive cells correlated with the recruitment of CD8^+^ T cells to the site of strongest IDO expression, which was paralleled by high expression of PD-L1, indicating a highly immunogenic situation modulated by cells with high IDO expression. Nevertheless, we need to consider that our cohort consists of patients who had received various mono- or combination therapies before resection of the investigated brain metastasis, possibly including pre-operative corticosteroid treatment, to minimize inflammatory side effects. Therefore, we are aware of the discussion that patient's therapy may affect the cellular component of immunoreactive populations, however, it has been shown that corticosteroids neither affect the TIL population nor the PD-L1 expression in melanoma brain metastases (38). Interestingly, IDO has also been considered for its negative impact by increasing the expression of FoxP3^+^ on regulatory T cells (71, 72), a correlation which we also observed in extracranial metastases. However, melanoma brain metastases do not appear to show this reciprocal interplay. The heterogenous IDO expression of melanoma metastases which we described here on the basis of immunohistochemistry, prompted our further investigation on the cell types that represent the major producers of IDO in melanoma brain metastases. Although melanoma cells *per se* expressed IDO, but at low intensity when compared to expression levels in immune cells, our data clearly indicate the impact of macrophages/microglia on IDO expression in melanoma brain metastases. Despite functional knowledge of myeloid cells, e.g., microglia and tumor-associated marcrophages (TAMs), in normal tissue, primary tumors and metastases, insights into their molecular identity, and clinical impact in intracranial metastases are still limited. In general, microglia and TAMs represent the most abundant non-neoplastic cells in brain metastases (73). Despite the lack of clinical data for the impact of microglia density and brain-associated TAM infiltration for patients prognosis, some pre-clinical data implicate tumor-promoting functions (74–76). In addition, functional charaterization of IDO expression with regard to polarization and functionality of both cell types in brain metastases are missing so far. Since our data are solely based on the use of FFPE specimens, the activity of IDO in the tumor mass with regard to tryptophan catabolism *per se* but also the impact of IDO for the activity and polarization of macrophages/microglia in the brain remain to be analyzed in fresh-frozen tissue samples from melanoma brain metastases but also in preclinical mouse models. For example, therapeutic intervention in the MT/ret-transgenic mouse model of metastatic melanoma would allow to analyse the population of IDO-positive TAMs/microglia in detail for their surface marker expression under IDO-targeted therapy. These newly identified surface markers could represent potential novel targets to reach more meaningful activity with regard to melanoma immunotherapy, potentially including re-education of macrophages as a new therapeutic strategy (77).

## Data Availability Statement

The datasets generated for this study are available on request to the corresponding author.

## Ethics Statement

Informed patient consent was obtained from all patients. The study was performed with approval by the ethics committee of the Medical Faculty, University Duisburg-Essen (ethics approvals no. 11-4715 and no. 15-6723-BO), and the ethics committee of the Medical Faculty, Heinrich Heine University Düsseldorf (ethics approval no. 5246).

## Author Contributions

IH, DS, and GR conceptualized and designed the study. DH-R and SM performed the experiments, generated, and analyzed data. SM and BB conceptualized and generated statistical analyses. ST-H, DP, AS, PJ, TS, JK, JF, KK, US, JR, GR, and DS collected and provided clinical material and data. GR and IH designed the selection of the patient cohort. IH conceptualized, coordinated, and directed the project. DH-R, SM, and IH wrote the manuscript. All authors performed manuscript review.

### Conflict of Interest

GR has received research grants from Roche and Merck, as well as honoraria for advisory boards from Abbvie. DS has received research grants from BMS and Novartis, as well as honoraria for being a member of advisory boards/consultant or as speaker from the following companies: Amgen, Array, Astra Zeneca, BMS, EMD-Serono, Incyte, Immunocore, Merck, Novartis, Philogen, Pierre Fabre, Pfizer, Regeneron, Roche, 4SC. The remaining authors declare that the research was conducted in the absence of any commercial or financial relationships that could be construed as a potential conflict of interest.
