# Supplementary figures and images for: Macrophages/Microglia Represent the Major Source of Indolamine 2,3-Dioxygenase Expression in Melanoma Metastases of the Brain
Source: Front Immunol. 2020 Feb 5;11:120. doi: 10.3389/fimmu.2020.00120 (PMC7013086; doi:10.3389/fimmu.2020.00120)

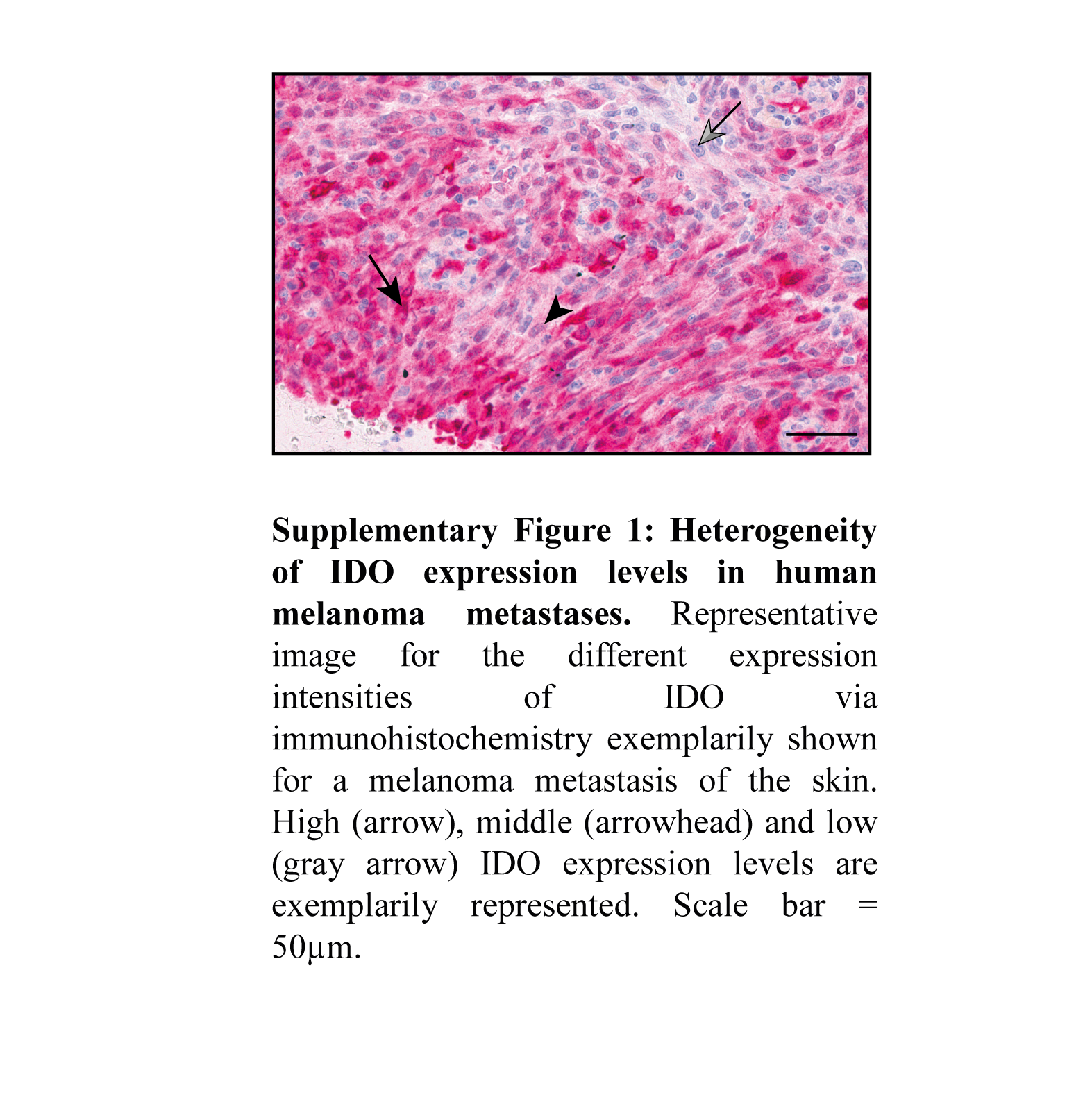

Supplement: Supplementary file 1 [file Image_1.TIF]

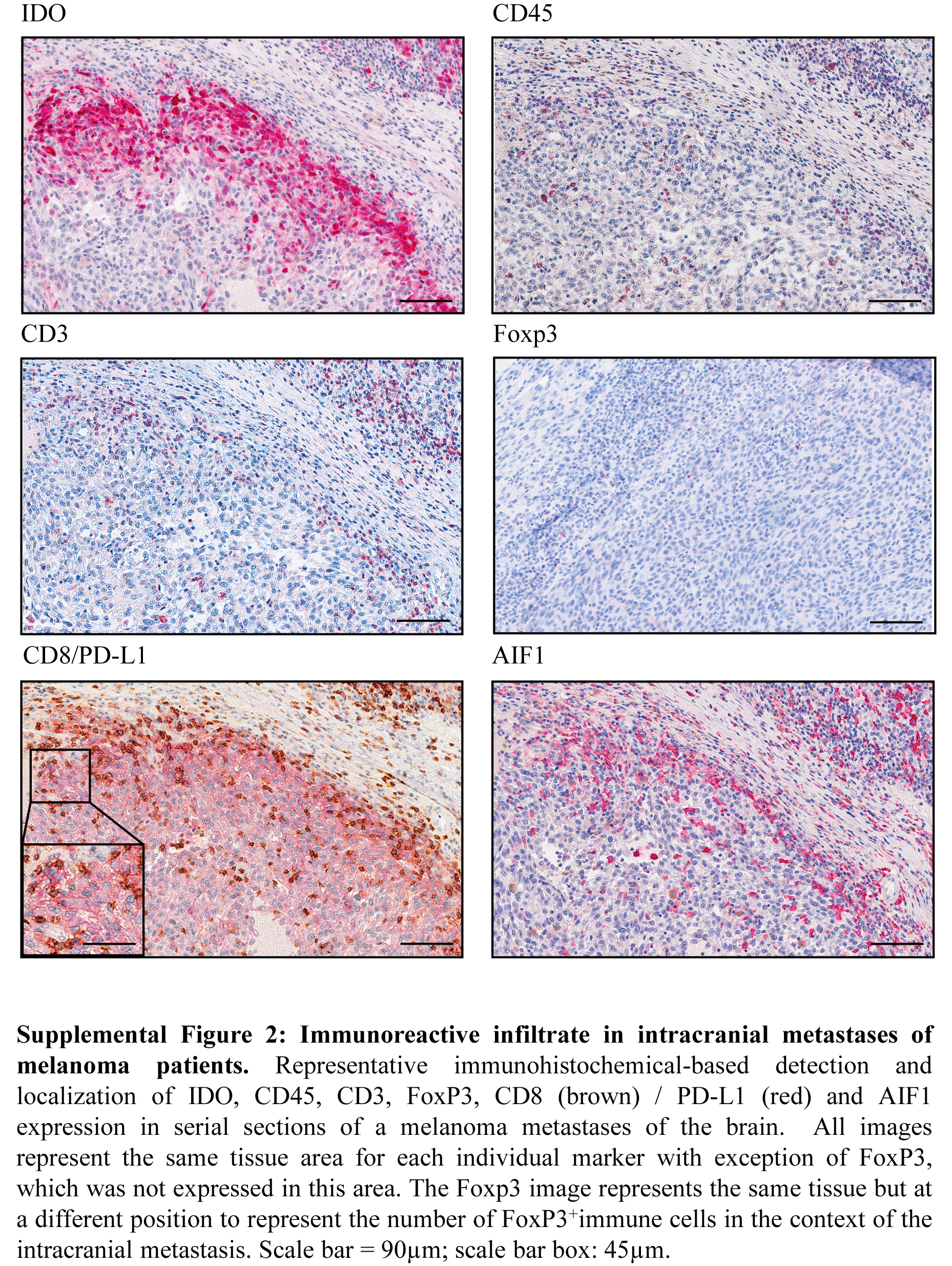

Supplement: Supplementary file 2 [file Image_2.TIF]

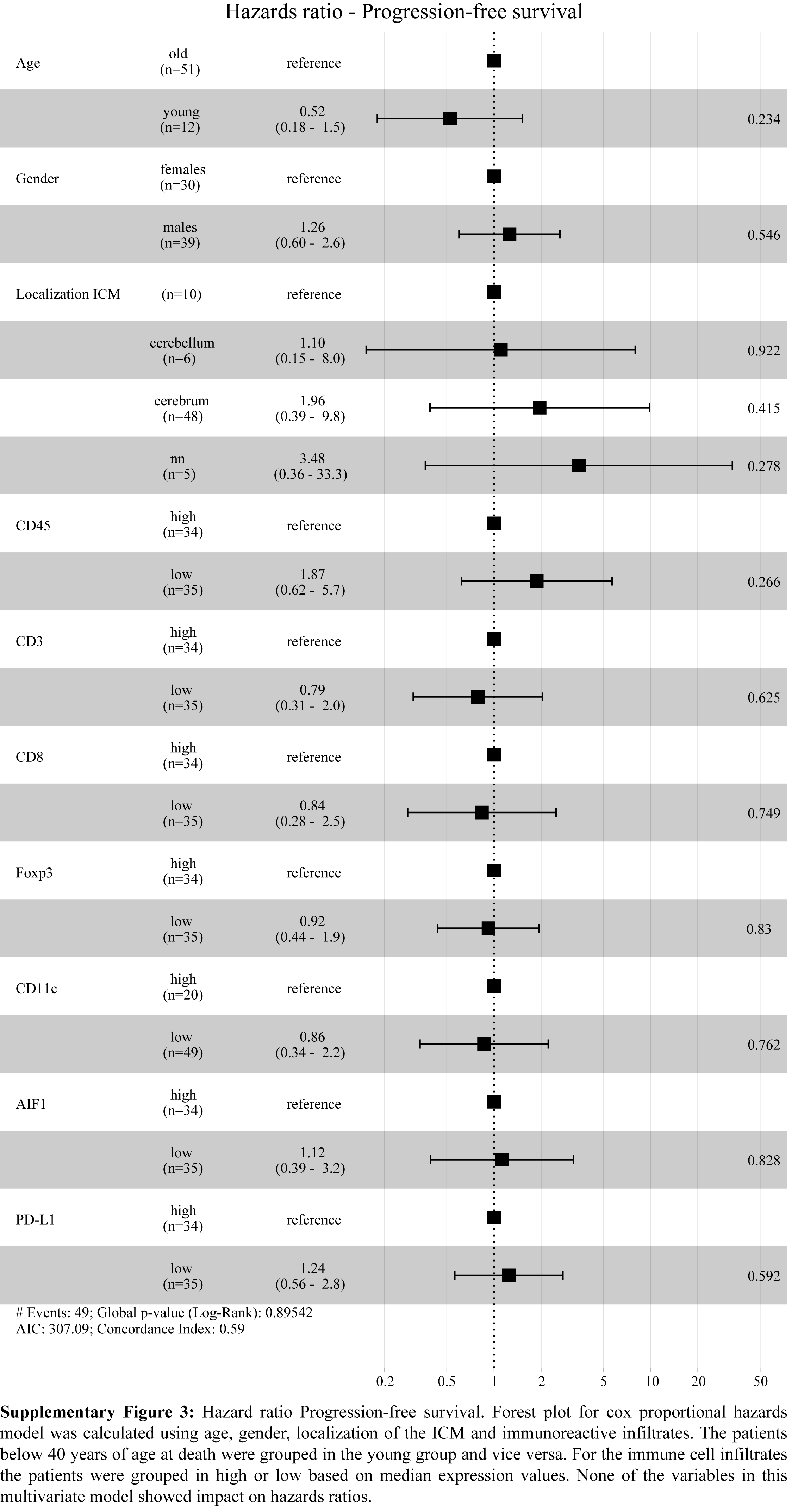

Supplement: Supplementary file 3 [file Image_3.TIF]
